# Recent Developments in Fluorescence Correlation Spectroscopy for Diffusion Measurements in Planar Lipid Membranes

**DOI:** 10.3390/ijms11020427

**Published:** 2010-01-28

**Authors:** Radek Macháň, Martin Hof

**Affiliations:** J. Heyrovský Institute of Physical Chemistry of ASCR, v.v.i., Dolejškova 2155/3, 182 23 Prague, Czech Republic; E-Mail: martin.hof@jh-inst.cas.cz

**Keywords:** lateral diffusion, fluorescence fluctuation spectroscopy, confocal microscopy, biomembranes, supported lipid bilayers, giant unilamellar vesicles

## Abstract

Fluorescence correlation spectroscopy (FCS) is a single molecule technique used mainly for determination of mobility and local concentration of molecules. This review describes the specific problems of FCS in planar systems and reviews the state of the art experimental approaches such as 2-focus, *Z*-scan or scanning FCS, which overcome most of the artefacts and limitations of standard FCS. We focus on diffusion measurements of lipids and proteins in planar lipid membranes and review the contributions of FCS to elucidating membrane dynamics and the factors influencing it, such as membrane composition, ionic strength, presence of membrane proteins or frictional coupling with solid support.

## 1. Introduction

Fluorescence correlation spectroscopy (FCS) was introduced in the early 1970s [1–3] and has been widely used for the determination of chemical and photophysical rate constants, local concentrations and, above all, translational and rotational diffusion coefficients of molecules and supramolecular complexes [4–15]. It is based on a statistical analysis of fluctuations of fluorescence intensity collected from a very small detection volume (1 μm^3^ or smaller), which is typically defined by the focus of a confocal or a multiphoton fluorescence microscope [16–18]. The diffusion of molecules in three dimensions was measured in the earliest FCS studies, but FCS investigations of lateral diffusion of molecules confined to 2-dimensional structures appeared soon afterwards. Nevertheless, while FCS became rapidly a standard and well established tool for 3-dimensional diffusion coefficient determination, fluorescence recovery after photobleaching (FRAP) was for a long time a preferred choice for measurements in two dimensions [19–21]. However, the single fluorophore sensitivity has made FCS an attractive alternative to FRAP, which requires high fluorophore concentrations, and motivated improvements in the method in order to overcome specific problems of FCS in two dimensions. Those are connected mainly to the accurate positioning of the detection volume with respect to the 2-dimensional sample and represent an important source of artefacts of standard FCS [22,23]. Especially in the last decade, several experimental approaches have been introduced, which overcome most limitations and sources of artefacts of standard FCS, making it a quantitative and reliable method for lateral diffusion measurements [24–29]. FCS has been applied in determinations of diffusion coefficients of molecules in planar systems including alumina membranes, surfactant bilayers or monolayers on air-water or oil-water interfaces and, above all, biological membranes, which have always represented the main motivation of the progress in FCS in two dimensions [22,30–36].

Apart from acting as barriers between the interior and the exterior of cells, biological membranes represent an environment required for many vital biochemical processes such as folding and activity of numerous proteins. A biological membrane is composed of proteins and lipids, a lipid bilayer being the key component of the membrane, which forms its structural matrix and provides mechanical stability and low permeability to ions and large molecules [37–39]. The biological significance of the lateral mobility of membrane constituents was introduced in the fluid mosaic model of Singer and Nicolson [40]. According to the model, all membrane components are freely diffusing along the plane of the membrane and the rate of their diffusion determines the kinetics of membrane-associated biochemical reactions. Later studies have shown that molecules are not distributed homogeneously in the membranes and that segregation driven by physical properties of the molecules can result in formation of domains differing in lipid and protein composition as well as in structural and dynamical parameters [37,41–44]. Their complexity of structure and dynamics initiated a further interest in investigation of biological membranes. The lateral diffusion coefficients of their constituents represent fundamental dynamical parameters of membranes and reflect their structure and, therefore, are in the focus of current membrane biophysics. Furthermore, the measured diffusion coefficients, which are readily accessible by a range of experimental techniques, can be confronted with values obtained by numerical simulations, thus helping to verify and refine the models of membrane structure on an atomistic scale [45–47].

The complexity of cellular membranes, where the diffusion of molecules is influenced by membrane inhomogeneities and interactions with cytoskeleton, imposes substantial difficulties on the interpretation of diffusion data [48–50]. Simplified artificial models of biological membranes are, therefore, widely used in order to establish a deeper understanding of the influence which membrane composition and structure have on the lateral mobility of its constituents. Among such model systems, planar lipid membranes are widely used, because they can be characterized by a wide range of experimental techniques and are very convenient for lateral diffusion investigations [51–53]. Planar lipid membranes may be divided into two main groups: free standing membranes, such as giant unilamellar vesicles (GUVs) [54–56], and membranes on solid supports, such as supported lipid bilayers (SLBs) [57–59]. Although a GUV is, strictly speaking, not planar, its large diameter (in the range of tens of μm) resulting in a negligible curvature allows diffusion measurements by the same experimental approaches, which are used in the case of supported membranes [60–62]. More details of the model systems and differences between them are discussed in Section 5.2.

This review explains the principles and specific problems of the lateral diffusion coefficient determination by FCS and reviews the state of the art in experimental approaches to FCS measurements in 2-dimensional systems. The progress in FCS investigation of lateral diffusion in planar lipid membranes is also reviewed and several key questions in the field are discussed; namely the difference between supported and free-standing membranes, the frictional coupling between the leaflets of a lipid bilayer or the influence of protein crowding on the rate of lateral diffusion.

## 2. FCS Measurement of Diffusion in Planar Systems

FCS extracts the information on molecular diffusion from the shape of the autocorrelation function *G* (τ) of the time trace of fluorescence intensity *I* (*t*) collected from a small detection volume within the sample. The autocorrelation function can be calculated by a hardware correlator or by software processing of the recorded time traces, the latter being more versatile and preferred in state of the art FCS [63]. The shape of the autocorrelation function reflects the timescale of fluorescence intensity fluctuations, which are caused mainly by the translational diffusion of fluorescent molecules in and out of the detection volume and by photochemical processes like intersystem crossing to a nonfluorescent triplet state. Fluctuations due to the lateral diffusion happen mostly on the millisecond to second timescale, while photochemical processes are usually much faster and their contributions can be, thus, separated [16,17,64]. The autocorrelation function *G* (τ) is defined by Equation (1), where the pointed brackets signify averaging over all values of time *t*:

(1) $$G(\tau )=\frac{〈I(t)\;I(t+\tau )〉}{{〈I(t)〉}^{2}}$$

In the case of a planar sample, the detection volume (or the detection area in this case), from which fluorescence intensity *I* (*t*) is collected, is defined by the intersection of the microscope focus and the plane of the sample. It can be approximated by a diffraction limited 2-dimensional Gaussian profile and the theoretical shape of *G* (τ) of a free Brownian diffusion is, then, given by Equation (2), where τ*_D_* (diffusion time) is the characteristic time a molecule spends in the detection area and *N* (particle number) is the average number of fluorescent particles (molecules, supramolecular complexes or quantum dots) within the detection volume [2,17,27,65]. Thompson introduces a geometrical factor γ into the expression for autocorrelation function; γ = ½ in the case of a 2-dimensional sample [65]. The factor γ changes the definition of particle number *N*, however, it does not affect the definition of diffusion time τ*_D_*:

(2) $$G(\tau )=1+\frac{1}{N}\frac{1}{1+(\tau /\tau_{D})}$$

If more fluorescent species exist in the sample, the contribution of the *i*-th species to the multicomponent autocorrelation function (3) is weighted by its fraction *F_i_* and its detection efficiency *Q_i_*, which depends on the quantum yield, lifetime and spectral properties of the fluorophore [65,66]:

(3) $$G_{M}(\tau )=1+\frac{\sum_{i=1}^{M}{\left(\frac{Q_{i}}{Q_{1}}\right)}^{2}F_{i}g_{i}(\tau )}{N{\left[\sum_{i=1}^{M}\frac{Q_{i}}{Q_{1}}F_{i}\right]}^{2}},\;\;\;\;\;g_{i}(\tau )=\frac{1}{1+(\tau /\tau_{Di})}$$

To account for fluorescence fluctuations caused by intersystem crossing, an average fraction of fluorophores in the nonfluorescent triplet state *T* and intersystem crossing relaxation time τ*_T_* need to be introduced to Equation (2) or analogically to Equation (3) for a multi-component system [13,67]:

(4) $$G(\tau )=1+[1-T+T\;\text{exp}(-\tau /\tau_{T})]\frac{1}{N(1-T)}\frac{1}{1+(\tau /\tau_{D})}$$

The interesting parameters τ*_D_* and *N* are extracted from the experimentally obtained autocorrelation function *G* (τ) via nonlinear fitting with an appropriate theoretical model (2)–(4) or by linear fit with a linearized form of the appropriate model [65,68]. An increasing number of components *M* results in a higher number of free parameters determined by fitting. That increases the probability that the fitted parameters are artefacts, because several combinations of parameters can describe the data well [69,70]. Several approaches have been proposed to estimate the accuracy of the parameters’ determination, which is rather complicated because of their highly nonlinear relation to the measured fluorescence fluctuations [71–74]. The signal-to-noise ratio in FCS is the highest when there is on average about one fluorescent molecule in the detection volume [22,75]. FCS is, therefore, counted among single molecule techniques, although the autocorrelation function always contains averaged contributions from a large number of molecules [25,76,77]. The optimal concentration of fluorescent molecules in the sample depends on the actual size of the detection volume. For the standard confocal setup it is usually in the nM range and in the case of planar samples it is in the range of single particles per μm^2^ and should not exceed 100 μm^−2^ [77]. A reduced size of the detection volume, which is attained for example in the case of two-photon FCS or several other special techniques [76,78–81], allows FCS measurements with higher fluorophore concentrations.

The excitation intensity for FCS should be adjusted carefully to reach sufficient molecular brightness (the average number of photons detected per fluorophore per unit of time) while minimizing the risk of artefacts due to photobleaching and optical saturation, which occurs when an increased number of molecules within the detection volume are not in the ground state but in an excited or triplet state, leading to a loss of proportionality between excitation and fluorescence intensities [16,17,82]. At the same time, the higher the molecular brightness, the higher the signal-to-noise ratio is and a tenfold reduction of excitation intensity would result in a need for approximately a hundred times longer measurement to reach a comparable statistical accuracy [75,82,83]. The optimum excitation intensity depends on the photophysical properties of the fluorophore under given conditions and on the time it resides in the detection volume (thus, on its diffusion coefficient and the size of the detection area) [84]. Assuming the values of photophysical parameters of typical organic fluorophores used in FCS, the excitation intensity should be kept well below 30 kW cm^−2^ [17,82]. Slowly diffusing molecules like large membrane proteins or molecules in gel phase domains of planar lipid membranes are especially sensitive to photobleaching, because they reside for longer times within the detection area [22,24,83,85–87]. Two-photon FCS suffers from stronger photobleaching and saturation effects in the focus of the microscope due to high intensities and pulsed excitation [83,88] (although it is known to cause less photobleaching outside the focus when compared to the standard confocal setup [76,83,89,90]). Several scanning approaches have been developed to overcome that problem [85,91–93] and will be discussed later in Section 2.3.

The diffusion time τ*_D_* represents a relative measure of lateral mobility of investigated molecules. To determine an absolute measure of molecular mobility, that means their lateral diffusion coefficient *D*, a characteristic length scale ω of the detection area must be known. The relation of *D* to τ*_D_* is, then, given by Equation (5). The surface concentration *c_S_* of the fluorophore can be calculated analogically from the particle number *N*:

(5) $$D=\frac{\omega^{2}}{4\tau_{D}}$$

The size of the detection area is typically defined by the radius of the diffraction limited waist of the laser focus, which means the minimal radial distance from the optical axis at which the laser intensity is *e*^2^ times lower than in its maximum [22,65,94]. The radius ω can be calibrated by an FCS measurement of diffusion of a reference fluorophore with a known value of diffusion coefficient. The measurement is usually performed in a solution of the reference fluorophore and τ*_D_* is found by fitting the measured autocorrelation function with a model for 3-dimensional diffusion assuming a 3-dimensional Gaussian shape of the detection volume [64,95]. Since the real shape of the detection volume differs from the assumed one (because of beam astigmatism, refractive index mismatch and other artefacts) and, furthermore, the radius of the beam-waist in the reference solution may vary from that in the sample of interest (because of differences in refractive index), the calibration procedure introduces errors to the FCS determination of *D* [16,17,22,25,96].

FCS of planar systems suffers also from errors caused by irreproducible axial positioning of the very thin sample (approximately 4 or 5 nm in the case of planar lipid membranes) within the detection volume, which can extend over a few μm in the axial direction [17,25]. If the plane of the sample does not coincide exactly with the waist of the focus, the divergence of the beam leads to a larger detection area and, thus, larger *N* and τ*_D_*. Positioning the sample by searching for the highest fluorescence intensity does not guarantee reproducibility, because the beam-waist does not necessarily coincide exactly with the highest fluorescence count rate [22]. The inaccuracies associated with external calibration and positioning may in sum lead to measured values of *D* varying by factor of 2 or larger [23]. Those problems motivated the development of several calibration-free FCS techniques, in which the need for an external calibration is avoided by an intrinsic ruler, which can be the precisely known distance between two foci in the 2-focus FCS [27,97], the distance between interference fringes in FCS with patterned illumination [98], the pixel size in camera-based FCS [77,99] or the step-size in *Z*-scan FCS [22] and various forms of scanning FCS [26,29,91,92].

### 2.1. 2-Focus FCS

This FCS technique uses the interfocal distance *d* as an intrinsic ruler [25]. Autocorrelation functions *G* (τ) for each focus and the crosscorrelation function *G_C_* (τ) between the two foci are calculated and fitted with appropriate theoretical models. In the case of Gaussian detection volumes, the crosscorrelation function *G_C_* (τ) is fitted with Equation (6), which assumes the autocorrelation functions described by Equation (2):

(6) $$G_{C}(\tau )=1+\frac{1}{N}\frac{\omega^{2}}{4D\tau +\omega^{2}}\text{exp}\left(\frac{-d^{2}}{4D\tau +\omega^{2}}\right)$$

Global fitting of individual autocorrelation functions and *G_C_* (τ) yields values of *D* and ω provided *d* is known. The accurate knowledge of *d* is essential, because the error in *D* scales quadratically with the error in *d* [27]. Measurement of fast diffusion requires a very small interfocal distance, which can be very precisely achieved using a Nomarski prism and two lasers with orthogonal polarizations creating two overlapping foci. Spatial crosstalk between the two foci is avoided by alternate pulsing of the lasers [27,100]. Two overlapping foci with a continuously adjustable interfocal distance can be generated using a Michelson interferometer [101].

The diffusion in membranes is often rather slow and larger interfocal distances *d* (in the order of 0.1 μm to μm) are sufficient. The signal from the two foci can be, then, simultaneously detected by different pixels of an electron multiplying charge-coupled device (EMCCD) placed in the image plane of the microscope. Although it is possible to detect the signal from the two foci using a dual-core optical fibre and two single point detectors [102], the setup based on an EMCCD is more flexible; it allows changing the interfocal distance (provided that a sufficient spacing between the pixels detecting signal from the two foci is maintained to minimize the crosstalk between them) and the same detection scheme is suitable also for measurements in more than two points simultaneously in order to perform cross-correlations between more pairs of foci [103–105]. Another experimentally simple method of 2-focus FCS uses a standard single focus laser scanning microscope and alternate scanning of two parallel lines [26,34,97].

### 2.2. Z-Scan FCS

The technique is based on measuring fluorescence autocorrelation functions *G* (τ) at different positions of the sample along the optical axis of the microscope (the *Z* axis) with a step-size typically of 100 or 200 nm, thus changing the distance Δ*_Z_* between the sample and the beam waist. Diffusion time τ*_D_* and particle number *N* exhibit a quadratic dependence on Δ*_Z_* [22,65,106] described by Equations (7), where λ is the wavelength of the excitation light in the medium of the sample and ω_0_ the *e*^−2^ radius of the beam waist. Parabolic fits of the measured dependencies of τ*_D_* and *N* on Δ*_Z_* with Equations (7) yield the physically relevant parameters *D*, *c_S_* and ω_0_ together with the exact *Z* position of the focus waist [22,65]:

(7) $$\tau_{D}(\Delta_{Z})=\frac{\omega_{0}^{2}}{4D}\left(1+\frac{\lambda^{2}\Delta_{Z}^{2}}{\pi^{2}\omega_{0}^{4}}\right),\;\;\;\;\;N(\Delta_{Z})=\pi \omega_{0}^{2}c_{S}\left(1+\frac{\lambda^{2}\Delta_{Z}^{2}}{\pi^{2}\omega_{0}^{4}}\right)$$

**Figure 1.**
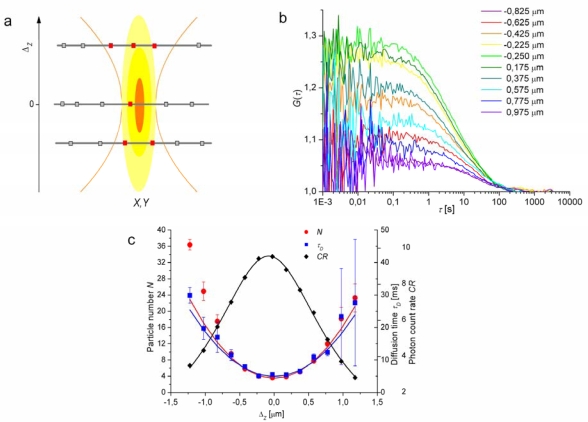
(a) A schematic illustration of the influence of the axial sample position on the size of the detection area and, thus, on *N* and τ*_D_* in Equations (2)–(7). (b) A set of autocorrelation curves obtained during a *Z*-scan measurement in a dilauroylphosphocholine (DLPC) SLB on mica. The bilayer was fluorescently labelled with β-C_8_-BODIPY 500/510 C_5_-HPC (Invitrogen, Carlsbad, CA) in lipid to dye ratio 10^5^; the details of the experimental procedures and instrumentation can be found elsewhere [107]. The values of Δ*_Z_* are given in the figure. (c) Δ*_Z_* dependencies of fluorescence intensity and parameters obtained by fitting of the autocorrelation functions. *N* (Δ*_Z_*) and τ*_D_* (Δ*_Z_*) are fitted with parabolas described by Equations (7); the photon count rate *CR* (Δ*_Z_*) (the fluorescence intensity) is fitted with a Lorentzian function, which approximates the axial intensity profile in the focus [13,108]. The resulting parameters are *D* = 3.4 μm^2^s^−1^; *c_S_* = 32 pmol m^−2^ and ω_0_ = 250 nm.

Figure 1 illustrates the principle of *Z*-scan FCS and shows an example of data measured in a SLB. Measurement of very slow diffusion by *Z*-scan FCS is limited by the temporal stability of the sample axial positioning, because autocorrelation curves have to be recorded in several positions defined with at least 100 nm accuracy [25].

Thermal undulations of free-standing membranes also add to the temporal instability [23]. Axial movements of the membrane can be, then, reflected in the autocorrelation curve as an apparent additional slow diffusion [25]. Deviations of τ*_D_* (Δ*_Z_*) and *N* (Δ*_Z_*) from the assumed parabolic shape (7) caused by distortions of the detection volume shape are other possible source of artefacts in *Z*-scan FCS.

### 2.3. Scanning and Imaging FCS

Slow diffusion, typically exhibited by large membrane proteins or by molecules in gel phase lipid bilayers, requires long measurement times to gain a sufficient statistical accuracy of the autocorrelation function (10^3^–10^4^ times longer than the relevant diffusion time τ*_D_* [25,94]). Long measurements are, however, prone to artefacts caused by photobleaching, saturation and instabilities in the experimental setup and the sample. Therefore, several scanning FCS techniques have been developed, which reduce the time for which slowly moving fluorophores are exposed to the laser beam and the overall time needed for the characterization of slow diffusion in membranes [8,29,92,109,110]. Instead of waiting for the fluorescent molecules to diffuse through the detection area, the focus of the microscope is moved with respect to the sample along a line [24,26,92,111] or a circle [29,91,112] and the residence time of the molecules within the detection area is, thus, decreased. Scanning FCS is, therefore, able to characterize the mobility of molecules with diffusion coefficients down to the order of 10^−3^ μm^2^s^−1^ [24]. Furthermore, the knowledge of the scanning speed (or the radius of a circular scan) circumvents the need for an external calibration [26,91,109].

Very slow diffusion in membranes can be investigated also by image correlation spectroscopy (ICS), which performs spatial correlations of images recorded by a laser scanning microscope [113]. Spatiotemporal image correlation spectroscopy (STICS) is an extension to ICS, which analyses both the temporal and spatial correlations and allows measurements of diffusion and flow velocities even in the presence of a significant fraction of immobile fluorophores [93]. Similar information is accessed by a related technique called k-space image correlation spectroscopy (kICS), which uses a transformation to the reciprocal space [109]. Reduction of the scanned area to a raster of points in raster image correlation spectroscopy (RICS) yields temporal resolution comparable to single-point FCS allowing investigation of rapid diffusion, while retaining the spatial information which contains information on slower dynamics. In this way, RICS can access a very broad dynamic range of molecular diffusion [28,114,115]. The detection of fluorescence by an EMCCD placed in the image plane of the microscope offers new possibilities in imaging FCS techniques. EMCCD detection offers the possibility to measure FCS simultaneously in more points of the sample, which is especially advantageous in setups where a large area of the sample is excited, such as in total internal reflection (TIR) microscopy (see Section 3.2) [103,104,109].

## 3. 2-Dimensional and 3-Dimensional Diffusion and Separation of Their Contributions

The detection volume of a confocal microscope, the most common FCS instrument, reaches up to a few μm in the axial direction [17]. It means that any fluorescent molecules present in the solution surrounding the planar sample can contribute to the detected fluorescence and, if they are present at high concentrations, can obscure the signal from the molecules diffusing in two dimensions. That problem is encountered for example in the cases when the fluorescent molecules (for example fluorescently labelled proteins) of interest partition only weakly to the membrane and an equilibrium between the membrane and the surrounding solution is established [116] or in studies on membranes of living cells, where structures such as endocytotic vesicles diffuse on a timescales similar to those of membrane lipids [25,94].

The contributions from molecules diffusing in the planar membrane and in the surrounding aqueous phase can be separated by fitting the autocorrelation function with a model containing contributions from both 2- and 3-dimensional diffusion [32,69,116], such as the one described by Equation (8). τ*_D_*_2_ and τ*_D_*_3_ are the diffusion times of molecules diffusing in two and three dimensions respectively; ω*_Z_* is the characteristic axial dimension of the detection volume:

(8) $$G(\tau )=1+[1-T+T\;\text{exp}(-\tau /\tau_{T})]\frac{1}{\left(1-T\right)}\left[\frac{A_{3}}{1+(\tau /\tau_{D3})}\frac{1}{{\left[1+\tau /\tau_{D3}{\left(\omega_{0}/\omega_{Z}\right)}^{2}\right]}^{\frac{1}{2}}}+\frac{A_{2}}{1+(\tau /\tau_{D2})}\right]$$

The amplitudes *A*_2_ and *A*_3_ of the contributions of two- and three-dimensional diffusion are related to the particle number *N* and the fraction *F*_2_ of molecules associated with the planar membrane via Equations (9), where β = *Q*_3_/*Q*_2_ is the ratio of detection efficiencies of a molecule in the aqueous phase and a molecule associated with the planar membrane. Donsmark and Rischel analysed the influence of an incorrect ratio β on the error in *N* and *F*_2_ [32]. Equations (9) simplify considerably in the case of identical detection efficiencies (β = 1) when *F*_2_ = *A*_2_ *N*. It is obvious that this approach also suffers from the problems with the positioning of the sample with respect to the focus and with the need for an external calibration. An improvement can be achieved by performing a *Z*-scan and fitting the individual autocorrelation functions with the model (8). The dependence of the amplitudes *A*_2_ and *A*_3_ on Δ*_Z_* is nontrivial and reflects the changes in the fraction *F*_2_ and in the ratio of the detection efficiencies β with the changing *Z* coordinate. Only the values of the amplitudes at a well-defined position when the membrane is in the focus (Δ*_Z_* = 0) are suitable for further quantitative considerations concerning the particle number *N* and the fraction of membrane-associated molecules *F*_2_ [116]:

(9) $$A_{2}=\frac{F_{2}}{N{\left(F_{2}+\beta +\beta F_{2}\right)}^{2}}\;\;\;\;\;\;\;\;\;\;\;\;\;\;\;\;\;\;\;\;\;\;\;\;A_{3}=\frac{\beta^{2}(1-F_{2})}{N{\left(F_{2}+\beta +\beta F_{2}\right)}^{2}}$$

### 3.1. Fluorescence Lifetime Correlation Spectroscopy and Lifetime Tuning

A different approach to filtering out the contribution of fluorophores, which are not associated with the planar membrane, is based on the shortening of fluorescence lifetime in the vicinity of a conducting surface and a method called fluorescence lifetime correlation spectroscopy (FLCS) [117–119].

**Figure 2.**
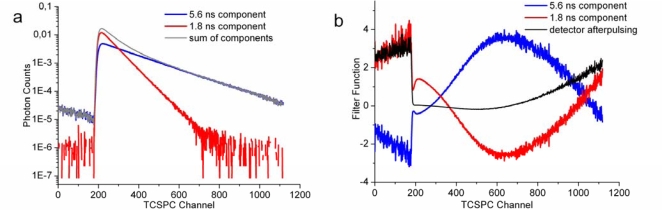
(a) TCSPC histograms of fluorescence decays of a sample containing a SLB on indium-tin oxide surface (lifetime 1.8 ns) and small vesicles in the aqueous phase (lifetime 5.6 ns). Both the SLB and the vesicles were labelled with β-C_8_-BODIPY 500/510 C_5_-HPC (Invitrogen, Carlsbad, CA); the details of the experimental procedures and instrumentation can be found elsewhere [117]. (b) The filter functions for the individual components in the sample and a function for filtering out the noise caused by detector afterpulsing. See the text for an explanation of the meaning and a discussion of the shape of the filter functions.

FLCS represents a synthesis of FCS with time correlated single photon counting (TCSPC). The experimental setup for FLCS requires a sub-nanosecond pulsed excitation (diode lasers being well suited for the purpose) and a possibility to record photon arrival times at two different timescales: relative to the excitation pulse with a picosecond resolution (TCSPC) and relative to the beginning of the measurement with a nanosecond resolution (FCS). TCSPC provides a fluorescence decay histogram, which is a superposition of decay histograms of all fluorescent species in the sample. If the decay characteristics of all components are known, their relative contributions can be extracted by a deconvolution of the measured histogram. Statistical filter functions can be, then, found, which, when applied to the measured decay histogram, recover the number of photons contributed by the individual components. Their role in FLCS is analogous to the role of optical filters in dual-colour FCS; they make it possible to statistically separate the contributions of the individual components photon by photon [118,119]. Figure 2 shows an illustration of the principle of FLCS in a sample containing two components with distinct fluorescence lifetimes (approximately 5.6 and 1.8 ns). The fluorescence decays are depicted in Figure 2 (a) and the corresponding statistical filters in Figure 2 (b).

Because the component with 1.8 ns lifetime has a higher intensity at the beginning of the decay, photons arriving shortly after the excitation pulse contribute with a higher weight (>1) to the autocorrelation function of the component with the shorter lifetime. At the same time their contribution to the autocorrelation function of the other component must be negative to ensure the unit total contribution of each photon. The photons with longer arrival times are, on the other hand, contributing more too the autocorrelation function of the component with 5.6 ns lifetime. Since the total number of photons arriving at longer times after the excitation pulse is lower, the absolute values of the filter functions are accordingly higher [119]. FLCS can be also applied to suppress noise in FCS data by using the differences between decay characteristics of the signal and the noise. In this way FLCS can filter out for example the contributions from scattered light, which have very fast decays, or the contributions from detector afterpulsing, which is manifested as a constant offset. If not removed, the contribution of detector afterpulsing leads to a distortion of the autocorrelation function at short values of lag time τ, which may be wrongly interpreted as triplet dynamics [119,120]. Figure 2(b) shows how the magnitude of the filter function for detector afterpulsing increases at longer times after the excitation pulse as a result of a decreasing number of arriving fluorescence photons (decreasing signal to noise ratio).

FLCS can be used also in the cases when the decay characteristics of only some of the fluorescent components in the sample are known. This is typically the case of a fluorescent molecule which partitions to the membrane and changes its lifetime upon the insertion to the membrane. It is possible to measure the fluorescence decay of the free molecule but not of the membrane-associated one, because of the equilibrium between the membrane and aqueous phase. Nevertheless, it is possible to filter out the contribution of the free fluorescent molecule with known decay and an autocorrelation function is obtained that corresponds only to the remaining components [121].

In many cases, however, the fluorophore does not change its lifetime upon association with the membrane or the change is too small to allow the separation of the components by FLCS. A method to increase artificially the difference in lifetimes between free and membrane-associated fluorophores was described by Benda *et al*. and, together with FLCS, it provides a rather universal approach to separate the contribution of fluorescent molecules in the aqueous phase from that of the molecules diffusing in the membrane [117]. The method is based on placing the planar membrane close to a conducting surface, which, due to strong charge transfer, shortens the fluorescence lifetime of fluorophores in its vicinity. The dependence of fluorescence lifetime on the distance form the surface can be quite steep; for example in the case of indium-tin oxide (ITO) the lifetime can change from zero to approximately a half of its maximal value within the first 10 nm from the surface. Figure 2 shows an example of such data: the component with a longer lifetime (approximately 5.6 ns) corresponds to a fluorescently labelled lipid in small vesicles in the aqueous phase and the other component (approximately 1.8 ns) to the same labelled lipid in a SLB on an ITO surface. The lifetime of the membrane-associated fluorescent molecules can be tuned by changing the distance of the membrane from the conducting surface by layers of a dielectric spacer such as silicon oxide [117,122–124].

### 3.2. Surface Confined FCS

Another solution to the problem with fluorophores in the aqueous phase lies in reducing the size of the detection volume in the axial direction. Such a reduction is usually reached by the confinement of the detection volume to a surface, which can be achieved by using evanescent waves in total internal reflection (TIR) FCS [80,125,126], surface generated fluorescence in supercritical angle (SA) FCS [127] or by confining the detection volume to optical nanostructures called zero-mode waveguides [78–80,128].

The total internal reflection occurs when light propagating through a medium of a higher refractive index *n*_1_ (*i.e.*, glass) encounters an interface with a medium of a lower refractive index *n*_2_ (*i.e.*, water) at an angle of incidence larger than the critical angle φ*_C_* = arcsin (*n*_2_/*n*_1_). No light propagates through the medium with the lower refractive index *n*_2_ and the fluorophores, located in the medium, can be excited only in the vicinity of the interface, where the exponentially decaying evanescent field reaches. The penetration depth of the evanescent field depends on the angle of incidence, wavelength of the light and refractive indices and can be well below 100 nm [80,125,126]. Incident angles larger than φ*_C_* are reached either by using a prism and collecting the fluorescence by a microscope objective opposite the prism or by illuminating an objective of a high numerical aperture with an annular ring of light and collecting the fluorescence by the same objective. The lateral dimensions of the detection volume are, however, rather large (in μm range) and a pinhole in the image plane is necessary for a lateral confinement of the detection area. Photobleaching of molecules located around the detection area is the main disadvantage of this technique [25,80,125]. That problem is solved by using a spatially resolved detection by an EMCCD, which collects the signal from a large part of the detection area. The approach, called imaging TIR FCS (ITIR FCS), offers similar information like STICS or RICS. An advantage of ITIR FCS is that measurements in many points of the sample are performed simultaneously. Both the temporal and spatial correlations can be analysed to gain the distribution of diffusion and flow velocities within the sample. The technique does not need any external calibration, because the well defined size of the pixels on the EMCCD serves as an intrinsic ruler [77,104,105].

Confinement of the detection volume in both the axial and lateral directions can be achieved by SA FCS, which is based on collecting exclusively the fluorescence emitted at angles above the critical angle. The construction of an objective for SA FCS and a detailed description of the method are given by Ries *et al*. [127].

Zero-mode waveguides are optical nanostructures which provide supreme lateral and axial detection volume confinement. Fluorescence is, in this case, excited by an evanescent field with short decay length (15–35 nm), which is confined to the bottom of sub-wavelength holes in a thin metal film on fused silica or glass [78–80,128,129]. Among the limitations of zero-mode waveguide FCS is the complexity of the autocorrelation function interpretation because of nontrivial conformations of the membrane within the nanostructures. A further limitation is the fact that more rigid membranes such as those in gel phase may not be able to invaginate into the nanostructures [79].

A similar reduction of the detection volume size in all directions can be reached also by using the near-field optical microscopy [80,130,131] or special nonlinear microscopic approaches which are capable of breaking the diffraction limit such as stimulated emission depletion (STED) [80,81,132]. Such nonlinear approaches are capable of detection volume confinement also in the case of freestanding membranes, which are not localized close to a specific surface. More information on the problematic of reduction of the detection volume in FCS can be found for example in the review [80] and references cited therein.

## 4. Deviations from Free Diffusion

The mean square displacement (MSD) of a molecule undergoing free Brownian diffusion in a 2-dimensional system satisfies the Einstein relation (10), where ***r*** is the position of the molecule and the diffusion coefficient *D* is a phenomenological constant dependent on the temperature and the microscopic properties of the tracer molecule and its environment. More exactly, this process driven by thermal fluctuations around the equilibrium should be called lateral self-diffusion to distinguish it from the diffusion driven by concentration gradients [133,134]:

(10) $$\text{MSD}=〈{\left(\text{r}(t)-\text{r}(0)\right)}^{2}〉=4Dt$$

Several studies of the diffusion in cellular membranes have found that the diffusion does not obey Equation (10), but its modification (11), where 0 < α < 1 is called anomalous exponent, *Γ* is a coefficient analogous to *D* and the diffusion is referred to as anomalous (or sometimes anomalous subdiffusion to indicate that smaller values of α correspond to slower movement of molecules) [133,135–137]:

(11) $$\text{MSD}=4\Gamma t^{a}$$

Theoretical studies have shown that anomalous diffusion can be a result of a broad distribution of jump times, correlations between diffusing particles or multiple diffusion rates and in cellular membranes it has been explained by lipid-protein binding interactions and by hindrance of diffusion by immobile proteins, lipid microdomains and cytoskeleton [133,138–141]. The deviation from free diffusion is also manifested by a different shape of the autocorrelation function measured by FCS and the anomalous exponent α has to be included in the theoretical model. The term τ/τ*_D_* in Equations (2)–(4) is, then, replaced by the term (τ/τ*_D_*)^α^ [32,142–144].

Theoretical and experimental analysis of diffusion in inhomogeneous systems has revealed how the characteristic length-scale of the measurement ω and its relation to the characteristic size of the obstacles influence the measured values of *D* [129,133,139,145,146]. The tracer molecule may perform a free diffusion locally; its diffusion is anomalous for intermediate values of ω and it can become normal again for large values of ω, but with a lower *D* than in the absence of obstacles. The transition to an apparent normal diffusion shifts to larger values of ω with increasing the fraction of area occupied by the obstacles [138,146]. The effect of mobile obstacles on the diffusion is less pronounced than that of immobile ones [133,147]. Therefore, when the size and concentration of obstacles are small enough with respect to ω, diffusion in inhomogeneous membranes seems normal. Nevertheless, even in such situations some information on the lateral organization of membrane inhomogeneities may be extracted from measurements of diffusion laws [142,148,149]. This approach is based on changing the measurement length-scale ω (and, thus, the detection area proportional to ω^2^) and analyzing the dependence of the diffusion time τ*_D_* on ω^2^. For sufficiently large values of ω (for which the diffusion appears normal), the dependence is linear and described by Equation (12) [129,142]:

(12) $$\tau_{D}=t_{0}+\frac{\omega^{2}}{4D_{\text{eff}}}$$

**Figure 3.**
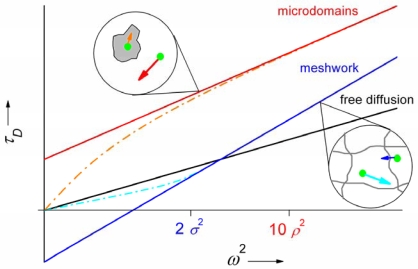
A schematic depiction of the diffusion laws (diffusion time τ*_D_* versus detection area ω^2^) for free Brownian diffusion and the two cases of hindered diffusion discussed in [142]: cytoskeleton meshwork and isolated microdomains with dynamic partitioning of tracer molecules. See text for details and meaning of σ and ρ.

The intercept *t*_0_ equals 0 in the case of free Brownian diffusion, but it can take non-zero values when the diffusion is hindered. The effective diffusion coefficient *D_eff_* is, then, different from the apparent diffusion coefficients measured at single values of ω. Wawrezinieck *et al*. [142] investigated two cases of hindered diffusion, which are likely to be encountered in the cellular membranes. The first system consisted of isolated lipid microdomains into which the tracer molecule can partition with certain probability and in which it undergoes a slower diffusion; the other system was an actin meshwork, which divides the membrane into corrals. The tracer molecule diffuses freely within a corral, but it needs a certain amount of energy to cross the barrier between the corrals. It has been shown that *t*_0_ is positive in the case of isolated microdomains and negative in the case of a meshwork. Furthermore, its magnitude bears information on dimensions of the obstacles. The diffusion is normal for ω^2^ > 10ρ^2^ or ω^2^ > 2σ^2^ where ρ and σ are the radius of the microdomains or the characteristic mesh size respectively [129,142,148]. The dependencies of τ*_D_* on ω^2^ (the diffusion laws) for free diffusion and the two cases of hindered diffusion are schematically depicted in Figure 3. Fully impermeable obstacles lead only to a reduction in diffusion coefficient and not to nonzero values of *t*_0_.

There exist several ways how to change the size of the detection area ω^2^ in FCS. For example Wawrezinieck *et al*. in the first work on FCS diffusion laws were changing the lateral extent of the excitation laser beam falling onto the back aperture of the objective to enlarge the detection area [142]. More recently authors from the same group applied zero-mode waveguides with aperture radii (and, thus, detection area radii) ranging from 75 to 250 nm [129]. Tuneable detection area radii in the same sub-wavelength size range are also achievable in STED FCS [81]. FCS methods using CCD cameras for fluorescence detection allow a very straightforward modification of the detection volume size by binning of adjacent pixels [77,99]. Variations of the detection area size are intrinsically present in *Z*-scan FCS. The size of the detection area during a *Z*-scan can be expressed as ω^2^ (Δ*_Z_*) = *N* (Δ*_Z_*)/*N*0, where *N*_0_ is the minimal particle number found by a parabolic fit of *N* (Δ*_Z_*) according to Equation (7). Humpolíčková *et al*. used the combination of *Z*-scan FCS and diffusion laws to compare the free diffusion of a fluorescent lipid analogue in a SLB with the diffusion of the same lipid analogue in the plasma membrane of living cells, where it was hindered by partitioning to microdomains (*t*_0_ > 0) [149].

## 5. FCS Elucidates Diffusion in Planar Lipid Membranes

### 5.1. Molecular Size versus Diffusion Coefficient

The size of a molecule is the most important parameter influencing its diffusion coefficient. Since lipids are the basic building blocks of biological membranes, the relation between size and diffusion coefficient is different for molecules comparable in size to lipids and molecules significantly larger, typically membrane proteins. The diffusion of lipids is usually described by the free area theory, which predicts for all molecules smaller or comparable in size to lipids the same value of *D* [150–152]. It is an oversimplification; however, it can be reasonably assumed that fluorescent tracer molecules similar to lipids in structure and membrane area they occupy mimic exactly the lipid diffusion. Such assumption is always made in FCS studies of lipid diffusion in membranes and the possible effect of the choice of the fluorescent tracer is usually not discussed. Typical examples of fluorescent tracers of lipid diffusion are fluorescent lipid analogues belonging to the family of long-chain dialkyl-carbocyanines, like DiD, DiI, DiA and DiO (Invitrogen, Carlsbad, CA) or lipids covalently labelled with fluorophores such as Bodipy, Rhodamine or Atto (Invitrogen; Avanti Polar Lipids, Alabaster, AL and Atto-TEC, Siegen, Germany). The validity of the above mentioned assumption is supported by findings of Kahya and Schwille [153] and Przybylo *et al*. [107], who found that the values of diffusion coefficient of DiD and both Bodipy headgroup- and tail-labelled lipids in SLBs on mica were equal within the experimental error. On the other hand, Chiantia *et al*. reported differences in the diffusion of several fluorescent tracers in SLBs on mica, which correlated with the electrostatic charge of the tracers. Positively charged tracers like DiD and DiO exhibited a slower diffusion than neutral Bodipy labelled lipids, suggesting that electrostatic interactions with the negatively charged mica surface may be responsible for the effect [97]. To verify whether the electrostatic interactions are indeed responsible for the differences in diffusion coefficient, measurements could be performed at different values of ionic strength, because the lower the ionic strength is, the more pronounced are the electrostatic interactions. However, it should not be forgotten that the changes in ionic strength can themselves modify the mobility of molecules in membranes, as has been for example shown in a study using neutral Bodipy labelled lipids [47] (see section 5.2.). Some problems can be encountered with Bodipy tail-labelled lipids. Bodipy moieties have been shown to prefer the polar region of the bilayer [154] and in tail-labelled lipids have, therefore, a tendency to loop back to the surface, in which way they perturb the local lipid order and increase the free area required for the tracer molecule [155].

The diffusion of molecules much larger than lipids is usually theoretically treated as a diffusion in a viscous continuum using the Saffmann-Delbrück approximation [152,156,157]. The theory predicts a weak logarithmic dependence of *D* on the radius of the molecule *R: D* ≈ ln(*R*^−1^), which makes it difficult to monitor by FCS small changes in *R* caused by protein dimerisation or altered geometry (such as helix tilting); only the formation of larger protein oligomers is reliably resolvable [158]. Recently Enderlein and co-workers observed by FCS in GUVs a *R*^−1^ dependence of *D*. They explain the deviation from Saffmann-Delbrück theory by the relatively small radius (only about twice that of a lipid) of the proteins under investigation; under which conditions the continuum model is no more applicable. Their finding is supported by a previous study [159] and is important for FCS investigation of membrane protein interactions. On the other hand for larger complexes, simulations predict a *R*^−2^ scaling of *D* [160].

Some proteins can move rather fast along the biological membranes thanks to their peripheral binding via a glycosylphosphatidylinositol (GPI) anchor. The membrane area occupied by the anchor is similar to that of a lipid molecule and the *D* of the GPI-anchored proteins is, therefore, similar to that of lipids as was shown by a FCS study of GPI-anchored green fluorescent protein diffusion in SLBs. Furthermore it was shown that the rigidity of the anchor is important for prevention of transient interactions of the protein with the underlying bilayer, thereby ensuring its rapid diffusion [161]. Molecules that do not insert into the membrane but only move along its surface, to which they are attracted by electrostatic forces, can exhibit an even faster diffusion than the membrane lipids as was observed in the case of a 13-residue basic peptide [162].

Increasing the concentration of membrane proteins slows down the diffusion of lipids and of the proteins themselves [152,163]. A recent *Z*-scan FCS study of bovine prothrombin binding to SLBs showed that prothrombin diffuses slower than the lipids, but the lipid and prothrombin diffusion coefficients decrease in a similar manner with an increasing prothrombin concentration. Furthermore, the difference in *D* between lipids and prothrombin is growing with an increasing content of dioleoylphosphoserine (DOPS) in the bilayer [116] indicating a specific interaction between prothrombin and DOPS [164]. Forstner *et al*. investigated the binding of cholera toxin subunit B to dimyristoylphosphocholine (DMPC) SLBs containing ganglioside GM1. The decrease in *D* was most pronounced close to the main phase transition of the lipids, when the crosslinking of GM_1_ by cholera toxin has the greatest impact on the lipid order [165]. A recent FCS study of protein diffusion in GUVs reported a linear decrease of the protein and lipid diffusion coefficients with an increasing protein concentration. The authors concluded that at protein densities ~25,000 μm^−2^ typical for biological membranes, the diffusion coefficients would be about an order of magnitude lower than the values measured in GUVs at protein density 3,000 μm^−2^ [158].

Binding of antimicrobial or cytolytic peptides to lipid membranes also affects the lipid diffusion coefficient in a manner dependent on the mechanism of interaction of the given peptide with membranes. The presence of the peptide molecules is not the only cause for the change in *D* since most antimicrobial and cytolytic peptides are known to create pores or other perturbations in the membranes [166–168]. Sheynis *et al*. reported a decrease in *D* caused by melittin and magainin II, but no effect of an artificial peptide KAL (KKA(LA)7KK), which corresponds to deeper insertion and smaller surface effects of KAL [169]. We have observed a decrease in lipid *D* to approximately 60% of its original value after treatment of a SLB with 1 μM melittin. The conclusion that pores are responsible for the large decrease is supported by a significant loss of lipids from the bilayer [170]. A large decrease in *D* was also observed in SLBs treated with 1 μM cryptdin-4 [171]. Removal of cryptdin-4 from the sample by washing it with an excess of a clean buffer resulted in a partial recovery towards the original values of *D*. Washing away melittin, however, did not change the *D* suggesting a difference in the membrane perturbations induced by the two peptides [170,171].

### 5.2. Supported versus Free-standing Planar Lipid Membranes

GUVs representing free-standing lipid membranes and being in size similar to cells are certainly the most realistic artificial model of plasma membrane of living cells. However, their preparation protocols are rather demanding and the most widespread ones are limited to low ionic strengths [54,55,172], although protocols allowing GUV preparation under physiological conditions have been also described [173]. SLBs are, on the other hand, a considerably less realistic model system, but very easy to prepare and stable and, thanks to their very well-defined geometry, accessible to characterisation by a wide range of experimental techniques [58,174–176]. They are formed on hydrophilic surfaces such as mica, glass, fused silica [51,175] or self-assembled alkanethiol monolayers [177,178] via adsorption and fusion of lipid vesicles [175,179,180] or via Langmuir–Blodgett and Langmuir–Schaefer techniques [59,181,182]. Although the lipid bilayer is separated from the solid surface by a thin aqueous layer (in the order of nm), thanks to which the bilayer retains its fluidity [183–185], the proximity of the support has a significant influence on the properties of the lipid membrane.

The first direct quantitative comparison of lipid diffusion in free-standing and supported lipid membranes was published by Przybylo *et al*. [107]. The relatively broad distribution of previously published values of lipid diffusion coefficients in GUVs (ranging from 3–6.5 μm^2^s^−1^ for the same lipid composition [87,153,186]) and SLBs (for example 2.6 or 4.2 μm^2^s^−1^ [22,187]) did not allow quantitative conclusions on the effect of the solid support on lateral lipid mobility. Apart from possible errors caused by inaccurate calibration in single-point FCS, the different experimental conditions such as ionic strength and sugar concentration are probably responsible for the incomparability of the individual results. While GUVs are usually investigated under very low ionic strengths (required in the typical preparation protocols) [55,60,188], physiologically more relevant conditions (100 or 150 mM NaCl) are common in SLB studies [22,97,136,170,187]. A decrease in lipid diffusion coefficient with an increasing concentration of NaCl has been observed both by FCS and molecular dynamics simulations [47,189]. Furthermore, GUVs are often stabilized by sugars such as glucose or sucrose [29,107,190,191]. FCS experiments and molecular dynamics simulations have shown slower lipid diffusion in the presence of various monosaccharides and disaccharides attributed to hydrogen bonding between a sugar molecule and phosphate groups of several lipid molecules [190,192,193]. Sucrose produces the strongest effect reducing the diffusion coefficient of lipids up to 3 times (at 1.5 M concentration) [190,193]. Przybylo *et al*. performed *Z*-scan FCS on GUVs and SLBs under identical conditions (150 mOsm glucose solution) and found that the lipid diffusion coefficient in DOPC GUVs *D_GUV_* = (7.8 ± 0.8) μm^2^s^−1^ is more than 2 times higher than in SLBs of identical lipid composition on mica with *D_SLB_* = (3.1 ± 0.3) μm^2^s^−1^. The finding is supported by the results of later studies [77].

Another limitation of SLBs is the fact that the very small distance between the proximal leaflet and the solid surface may prevent correct reconstitution of transmembrane proteins into the bilayer. To increase the space available on both sides of the membrane while maintaining the convenient geometry of SLBs, membranes on soft polymer layers (polymer-cushioned bilayers) [180,194–196] or linear polymer spacers covalently coupled to lipid head groups (polymer-tethered bilayers) [197–200] have been developed. Polymer-tethered bilayers were used for example in a recent scanning-FCS study of G protein-coupled receptor diffusion [201]. However, the tethered lipids may act as obstacles and hinder the diffusion in the planar membrane [146,198]. An alternative free-standing planar lipid membrane can be prepared by spreading a bilayer over an aperture (40–150 μm in diameter) in a polytetrafluoroethylene septum. The diffusion coefficient of lipids in such a model membrane determined by FCS (8.1 ± 0.4) μm^2^s^−1^ corresponds to the values measured in GUVs [202].

### 5.3. Inter-leaflet Coupling and Membrane Asymmetry

The membranes of living cells are known to be asymmetric; the two leaflets of the membrane differ in their lipid and protein composition and they also face different aqueous phases [203,204]. It is, therefore, very interesting to know how strong is the interaction between the membrane leaflets and how are the dynamic properties of one leaflet related to those of the other. Such questions are of a high relevance for biology; namely the question of how do the structural and dynamic parameters of the cytosolic leaflet of the plasma membrane reflect the changes in the outer leaflet induced by lipid phase separation or peripheral binding of other molecules to the membrane.

In terms of artificial lipid membranes, the question of inter-leaflet coupling was addressed by Przybylo *et al*., who concluded that a strong inter-leaflet coupling exists in SLBs and lipids in both leaflets diffuse with the same velocity [107]. The argumentation was based on the approximately 2-fold difference in lipid diffusion among supported (SLBs) and free-standing bilayers (GUVs). In the absence of a strong inter-leaflet coupling, the lipids in the distal leaflet should diffuse like lipids in GUVs and the lipids in the proximal leaflet would have to be approximately 4 times slower. Since FCS can reliably distinguish contributions from molecules which differ at least 1.6–2 times in their diffusion coefficients [77,205], such a large difference between proximal and distal leaflet would result in two distinct values of diffusion time measured in SLBs. The measured autocorrelation curves could be, however, fitted successfully with a model containing a single diffusion time, indicating, thus, a strong inter-leaflet coupling. Zhang and Granick arrived to the same conclusions when they selectively quenched the fluorophores in the distal leaflet by iodide [68,187,206]. They acquired evidence for a strong inter-leaflet coupling in SLBs on quartz prepared both by vesicle adsorption and fusion and by Langmuir-Blodgett technique. The same effect was reproduced in SLBs on polymer cushions [206] and the strong inter-leaflet coupling was also found in experiments when the diffusion in the distal leaflet was slowed down by polymer binding [68,187].

A recent FCS study investigated the diffusion of poly-lysine in free-standing planar membranes and its influence on lipid diffusion. *D* for both poly-lysine and lipids decreased with the number of lysine units in the polymer and an evidence for strong inter-leaflet coupling of poly-lysine diffusion was found, indicating that two poly-lysine molecules on the two leaflets of the bilayer move together with lipids sandwiched between them forming a nanodomain [202]. Such alignment of lipid domains is likely to play an important role in transmembrane signalling.

## 6. Concluding Remarks

FCS methodology for investigation of the lateral mobility of molecules in planar systems has undergone a rapid development in the past decade, which has been motivated mainly by the interest in mobility of molecules in biological membranes and their artificial models. The currently available FCS approaches, which include for example *Z*-scan FCS, 2-focus FCS or many variants of scanning FCS and imaging FCS, have overcome all the important limitations encountered originally in FCS measurements on planar samples. They have solved the problem with exact positioning of the planar sample into the focus of the microscope and they do not need external calibration. Some of the methods, such as RICS, can access a very broad range of diffusion coefficients, practically the whole range of diffusion coefficients of molecules in lipid membranes. Other methods, such as TIR FCS or FLCS with lifetime tuning, are capable of suppressing a fluorescence background not originating from the planar sample of interest, while yet other techniques can achieve a considerable reduction of the detection volume in all directions and, thus, allow FCS measurements with a high spatial resolution and at higher concentrations of fluorescent tracer molecules. To conclude, state of the art FCS methodologies represent a versatile and efficient tool for investigation of diffusion (and other dynamic processes) in lipid membranes. They combine single molecule sensitivity with reasonably short measurement times acceptable for routine essays. Furthermore, all the FCS methods described here can be used for investigation of membranes of living cells or even multicellular organisms [207] and can, therefore, help to relate the molecular diffusion in model and native membranes. It is certain that with such potential, FCS will help to bring answers to many open questions of current membrane biology and biophysics.
